# Asymmetric presentation with a novel RP2 gene mutation in X-Linked retinitis pigmentosa: a case report

**DOI:** 10.1186/s12886-023-02968-4

**Published:** 2023-05-17

**Authors:** Hyun Woo Lee, Eun Kyoung Lee

**Affiliations:** 1 Pre-medical Program, Seoul National University College of Medicine, Seoul, Korea; 2 Department of Ophthalmology, Seoul National University College of Medicine, Seoul National University Hospital, #101, Daehak-ro, Jongno-gu Seoul, Republic of Korea

**Keywords:** Retinitis pigmentosa, Multimodal imaging, *RP2* Gene, X-linked retinitis pigmentosa

## Abstract

**Background:**

We present the detailed multimodal imaging analysis in a case of X-linked retinitis pigmentosa (XLRP) exhibiting a markedly asymmetric presentation with a novel *RP2* mutation.

**Case presentation:**

A 25-year-old woman complained of decreased vision in the right eye as well as night blindness. Her visual acuity was 20/100 (OD) and 20/20 (OS). Fundus examination revealed bone spicule pigmentation with tessellated changes in the fundus within the posterior pole. Optical coherence tomography (OCT) showed generalized disruption of foveal microstructures in the OD. No abnormal findings were identified, but localized ellipsoid zone band losses were observed on OCT in the OS. Fundus autofluorescence revealed multiple patchy hypo-autofluorescent lesions in the OD and a tapetal-like radial reflex against a dark background in the OS. Fluorescein angiography and OCT angiography revealed diffuse mottled hyperfluorescence with reduced retinal vessel density in the OD and no evidence of vascular compromise in the OS. Goldmann perimetry demonstrated a constricted visual field, and electrophysiological assessment revealed an extinguished rod response and a severely impaired cone response in the OD. Molecular genetic tests via next-generation sequencing revealed the pathogenic variant to be a heterozygous frameshift mutation in *RP2* (*RP2*, p.Glu269Glyfs*7), resulting in premature termination of the protein.

**Conclusions:**

Random X-inactivation may be attributed to interocular differences in the severity of XLRP in female carriers. A novel frameshift mutation in the *RP2* gene and a comprehensive phenotypic evaluation in the current study may broaden the spectrum of the disease in XLRP carriers.

**Supplementary Information:**

The online version contains supplementary material available at 10.1186/s12886-023-02968-4.

## Background

Retinitis pigmentosa (RP) is the most common inherited retinal disease and is characterized by the progressive degeneration of the rod and cone photoreceptors. The classic triad symptoms of RP are pale waxy optic disc, attenuation of retinal vessels, and bone spicule pigmentation [1, 2]. As the retinal pigment epithelium (RPE) and photoreceptor degeneration progresses, nyctalopia, gradational vision loss, and constriction in the visual field develop, causing subsequent irreversible vision loss [3]. The disease is genetically heterogenous, and it has been linked to nearly 100 different genes [4, 5].

X-linked retinitis pigmentosa (XLRP) is particularly severe, with an early onset in childhood and rapid progression. Retinitis pigmentosa GTPase regulator (*RPGR*) gene (OMIM #312610) variants account for 70 − 80% of XLRP, *RP2* (OMIM #312600) variants account for a further 5 − 20%, and *OFD1* (OMIM #300170) has been identified as a rare cause of XLRP [6–8]. Mostly, genetic RP presents bilaterally, although some cases show interocular asymmetry. Female carriers of XLRP show variable clinical symptoms of the disease with asymmetric manifestations [9]. The wide spectrum of phenotypes in female carriers of XLRP is likely attributable to random X chromosome inactivation during embryogenesis [10]. This random inactivation, a physiological phenomenon called lyonization, persists in daughter cells and results in a mosaic distribution [10–12].

The human RP2 protein comprises of 350 amino acids and is widely expressed. The function of RP2 is not fully understood, but it is widely considered to constitute the plasma membranes of all retinal cell types, and that the acylation of RP2 is critical for its function in the retina [13]. The encoded RP2 protein is implicated in ciliary trafficking of myristoylated and prenylated proteins in photoreceptor cells. In the present study, we identified a novel RP2 mutation in a female XLRP carrier with a markedly asymmetric presentation. However, little is known regarding the relationship between RP and interocular asymmetry. Since the number of reported case series of asymmetric RP is limited, the elucidation of this condition requires more information. Herein, we present the detailed multimodal imaging analysis in an RP case showing a markedly asymmetric presentation and a previously unreported null mutation in the *RP2* gene.

## Case presentation

**Fig. 1:**
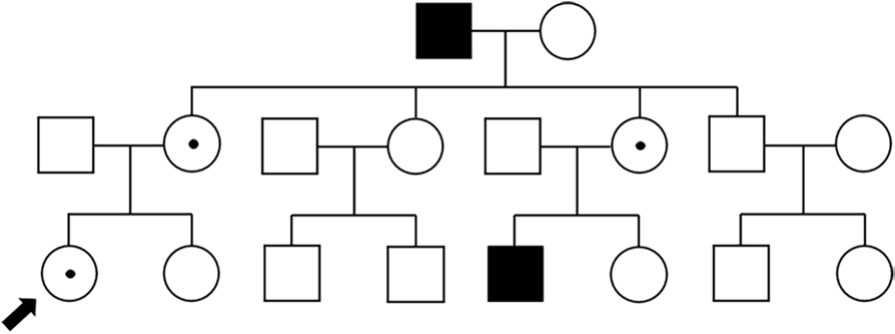
The family pedigree is shown. Open symbols indicate unaffected family members; the closed symbol, the individual who reported nyctalopia; and circle with a dot in the center, a female carrier. The index patient is indicated by the arrow

A 25-year-old woman presented to our clinic with decreased vision in the right eye as well as night blindness. The patient was systemically healthy and had no history of ocular trauma or uveitis. She could not easily navigate a dark movie theater without support since high school, but it did not worsen, and the condition was maintained. She reported a family history of RP with poor vision and nyctalopia involving her maternal grandfather and maternal cousin. However, we were unable to ophthalmologically examine for her family members. The family pedigree based on history and symptoms is shown in Fig. 1.

**Fig. 2:**
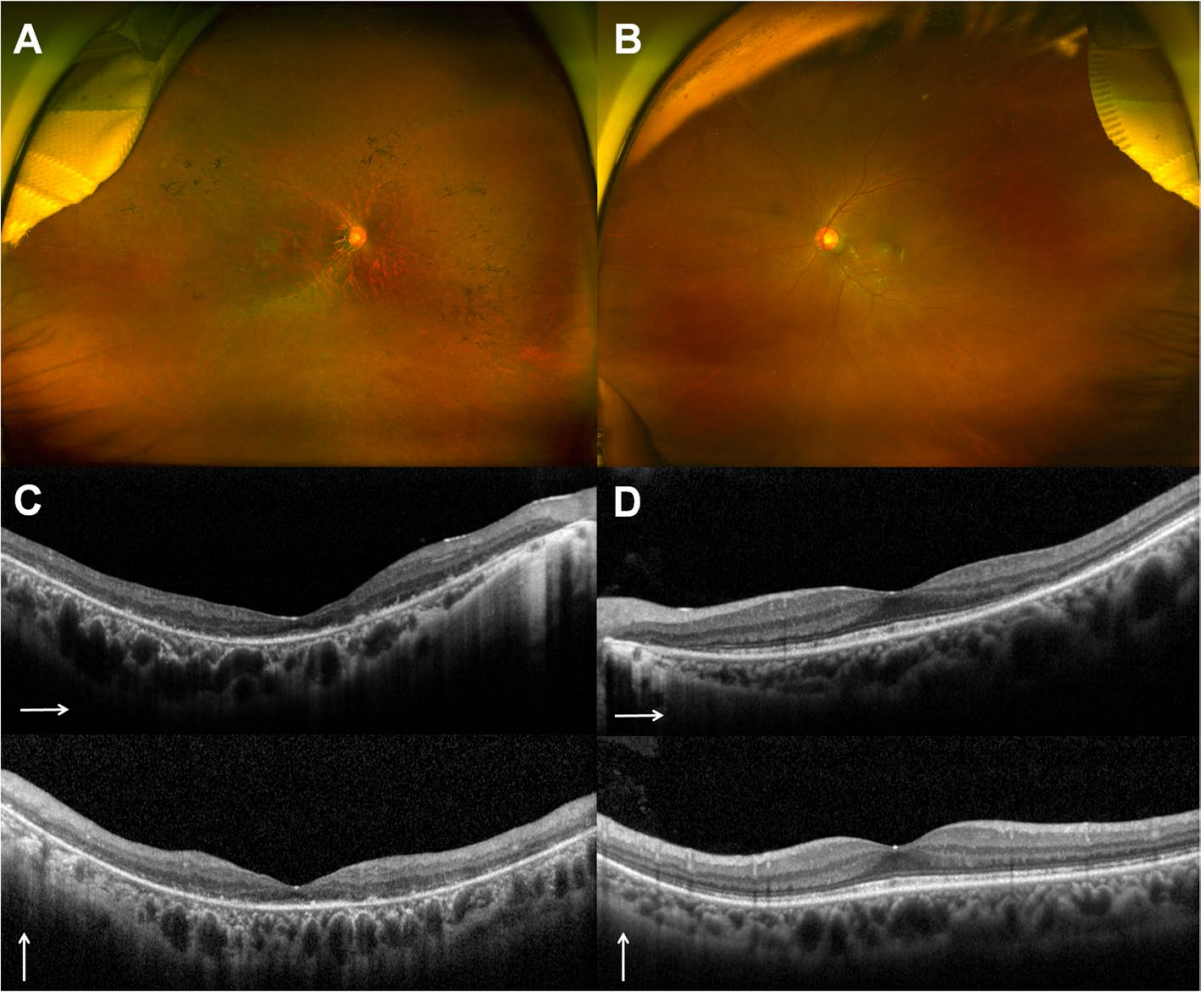
Ultra-widefield fundus photographs (**A, B**), and optical coherence tomography (OCT) images (**C, D**). **A** Bone spicule pigmentation, attenuation of retinal vessels, and tessellated fundus changes within the posterior pole are noted in the right eye. **B** No conspicuous bone spicule pigmentation is identified in the left eye. **C** Generalized disruption of foveal microstructures with a faint preserved residual ellipsoid zone in the superior macular area as well as subfovea in the right eye are noted. **D** Increased reflectivity from the retinal pigment epithelium and localized attenuation and losses of the ellipsoid zone band are identified in the left eye. Long thin white arrows indicate OCT scan direction

**Fig. 3:**
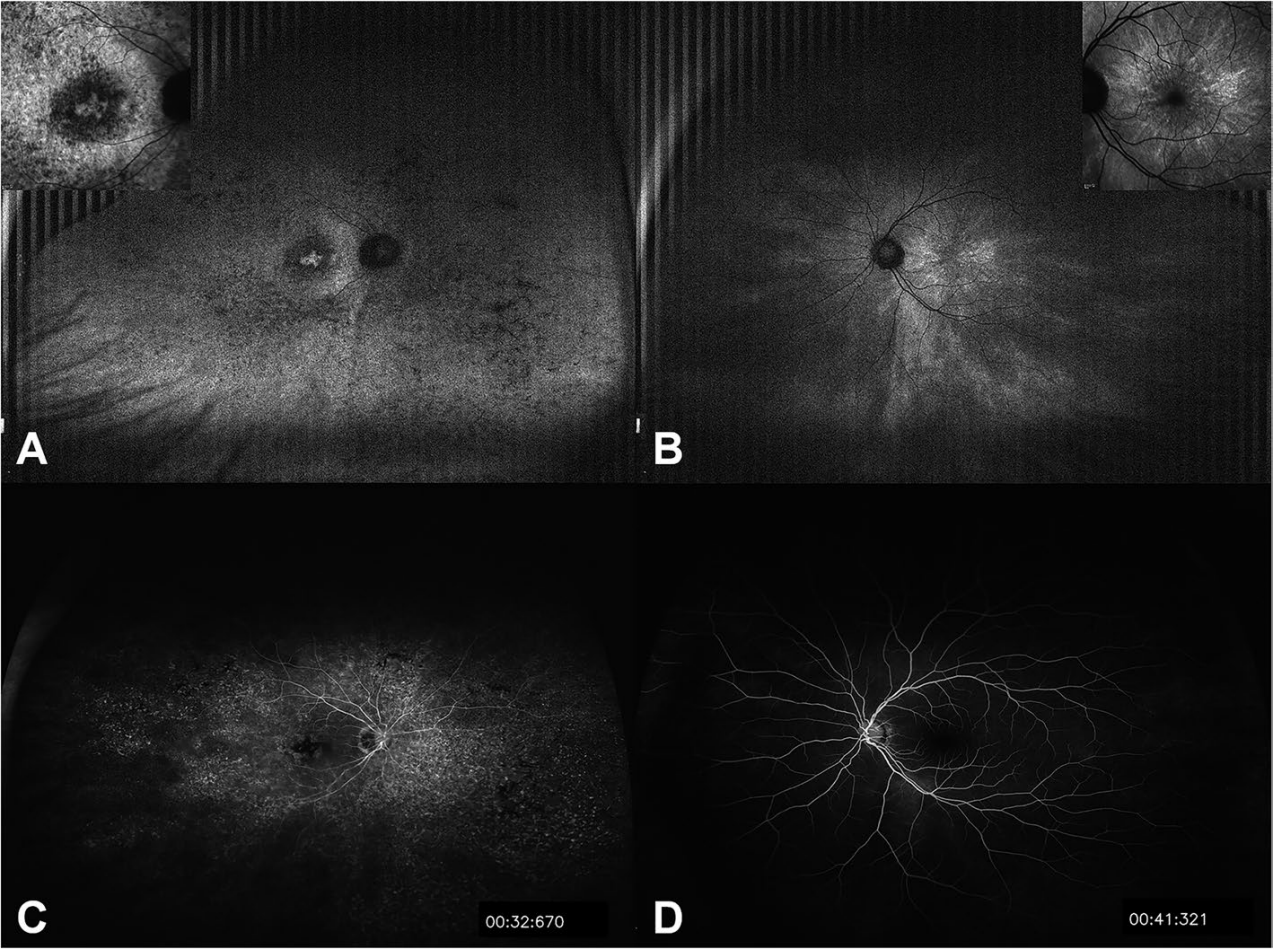
Fundus autofluorescence (FAF) (**A, B**) and ultra-widefield fluorescein angiography (FA) images (**C, D**). **A** Multiple patchy and reticular hypoautofluorescent lesions with an abnormal hyperautofluorescence in the fovea exists in the right eye. **B** Tapetal-like radial reflex against a dark background was noted in the left eye. The top corner shows the macular FAF images with completely different aspect of FAF findings. **C** Mottled hyperfluorescence corresponding to the affected retinal areas are noted. **D** No specific abnormalities were identified

**Fig. 4:**
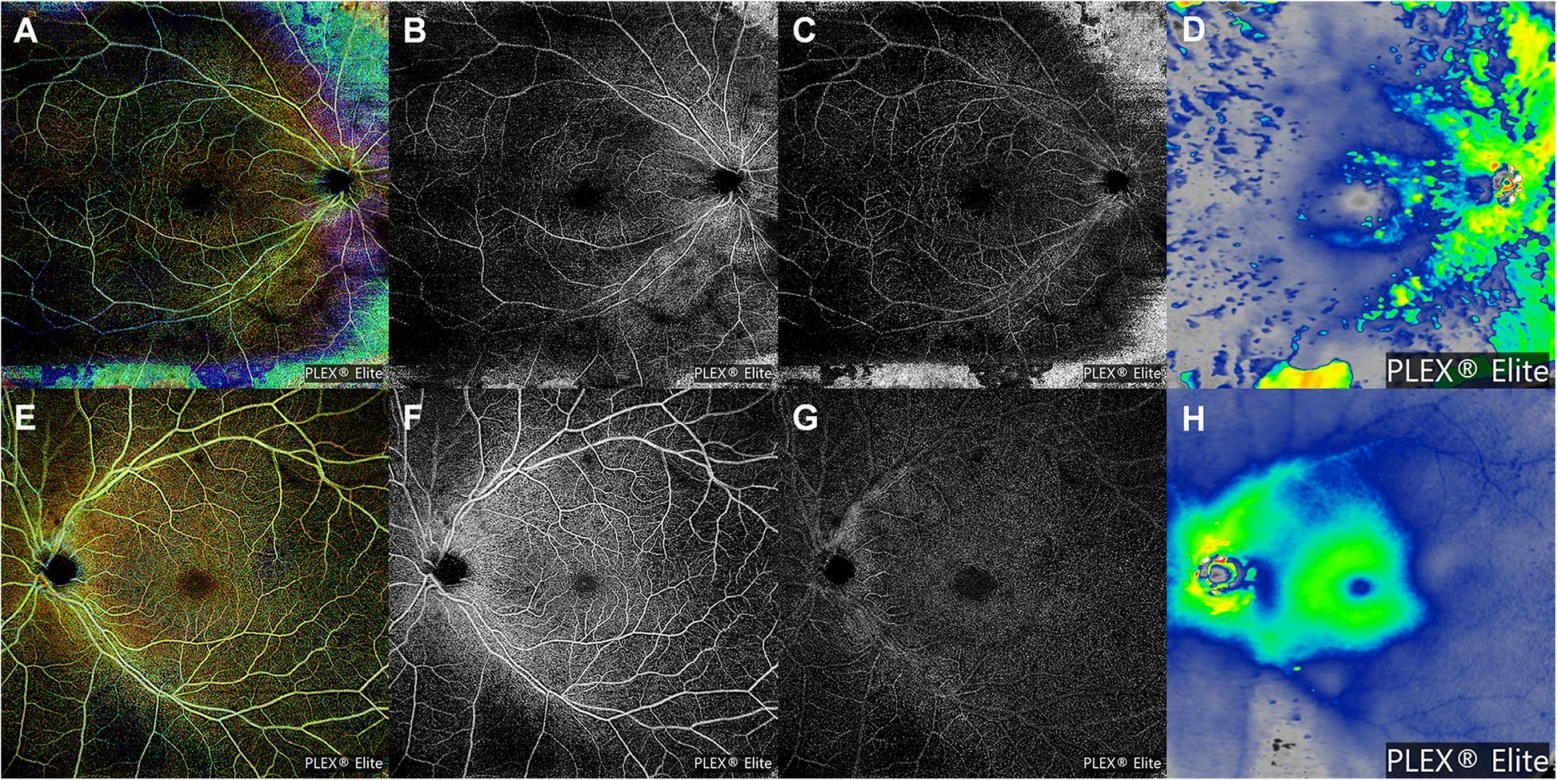
Swept-source optical coherence tomography angiography images (12 × 12 mm). These correspond to the retina depth-encoded (**A, E**) superficial capillary plexus (**B, F**) deep capillary plexus slab (**D, G**) and retinal thickness map (**D, H**)

On examination, the best-corrected visual acuity was 20/100 in the right eye (OD) and 20/20 in the left eye (OS). Her refractive error was -4.0 Dsph = -4.0 Dcyl x A180 OD and -1.25 Dsph = -3.25 Dcyl x A20 OS. A slit-lamp examination revealed no specific findings. Fundus examination revealed multiple bone spicule pigmentations and attenuation of retinal vessels in the OD. Tessellated fundus changes within the posterior pole were also observed. However, no conspicuous bone spicule pigmentation was found, with an almost normal appearance of the retina in the OS (Fig. 2A, B). Optical coherence tomography (OCT) revealed visible thinning of the entire outer retina and generalized disruption of foveal microstructures, with a small area of preserved faint ellipsoid zone subfoveally in the OD. Localized attenuation and losses in the ellipsoid zone band were observed in OS (Fig. 2C, D). Fundus autofluorescence (FAF) revealed multiple patchy and reticular hypoautofluorescent lesions with abnormal hyperautofluorescence in the fovea of the OD. Interestingly, a completely different aspect of the FAF findings was noted in the OS: a tapetal-like reflex showing a characteristic bright radial reflex against a dark background (Fig. 3A, B). Ultra-widefield fluorescein angiography (FA) imaging showed diffuse, blotchy, or mottled hyperfluorescence corresponding to the affected whole retinal areas in the OD. No specific abnormalities were identified in the OS (Fig. 3C, D). Optical coherence tomography angiography (OCTA) revealed significantly reduced retinal vessel density, blood flow, and retinal thinning in the OD (Fig. 4).

**Fig. 5:**
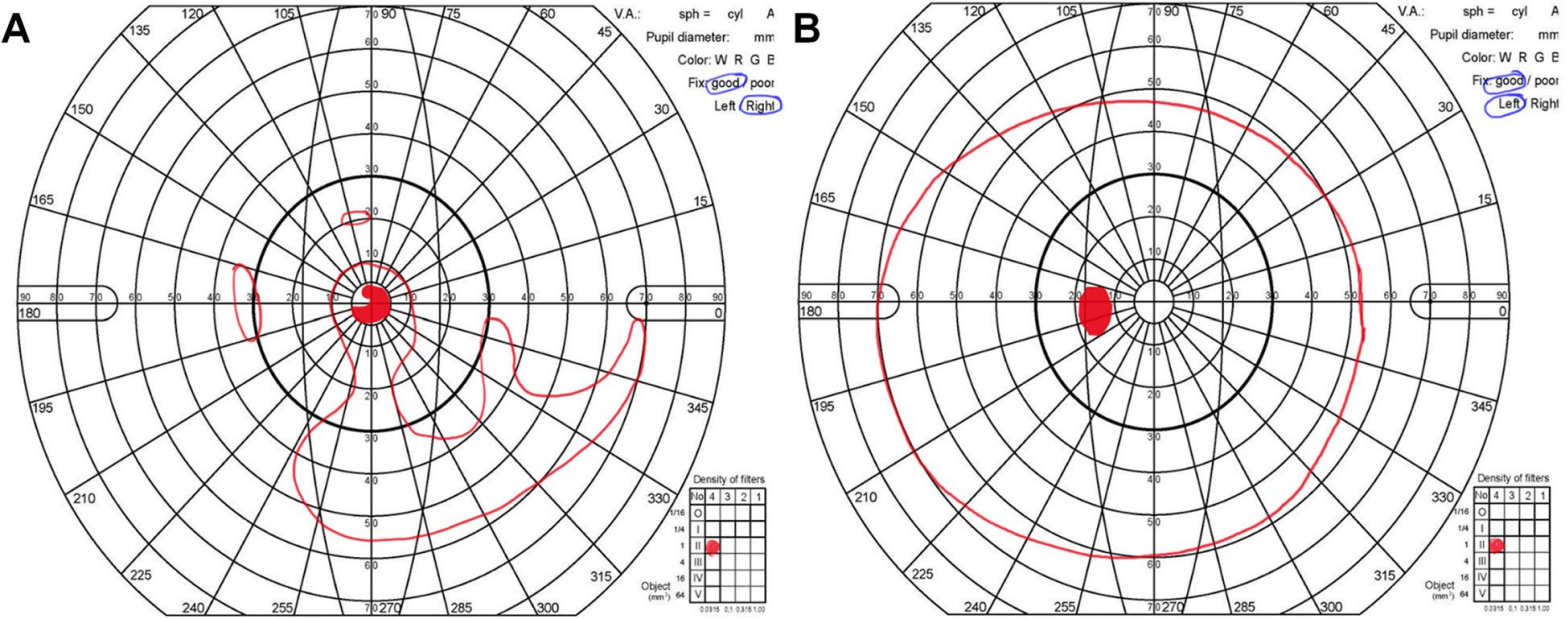
Goldmann perimetry examination. **A** Constricted visual field with the remaining 10° central visual field in the right eye. **B** No specific abnormal findings in the left eye

**Fig. 6:**
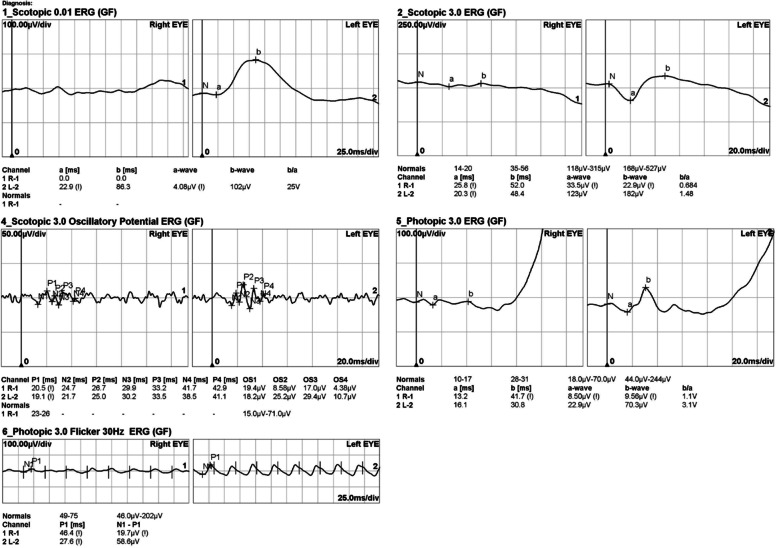
Electroretinography examination. Extinguished rod response and a severely impaired cone response are noted in the right eye. The amplitudes of rod and cone response showed unremarkable findings in the left eye

Goldmann perimetry demonstrated a central visual field within 10° and islands of the visual field, particularly on the inferior side of the OD. A central scotoma was also identified in the remaining central visual field. A normal visual field with a physiological blind spot was observed in the OS (Fig. 5). Electrophysiologic assessment was performed, and electroretinography (ERG) revealed an extinguished rod response and a severely impaired cone response in the OD. The amplitudes of the rod and cone responses were unremarkable in the OS, revealing marked asymmetry in both the structure and function of the retina (Fig. 6).

Molecular genetic tests using next-generation sequencing (NGS)-based gene panels were performed using peripheral blood samples obtained from patient after providing informed consent. The exome-based targeted panel comprised 244 candidate genes associated with inherited retinal diseases and screened the coding region and its flanking region of the gene using the NovaSeq system (Illumina, USA) (Supplementary Table). The variant interpretation was performed using the guidelines of the American College of Medical Genetics and Genomics (ACMG) [14]. We identified a novel *RP2* mutation (c.803dup) in exon 3 at position 803 that caused a frameshift and premature termination signal at codon 269 (*RP2*, p.Glu269Glyfs*7, heterozygote). These changes have not been previously reported in the literature. This corresponded to the carrier of XLRP2, and no other pathogenic or likely pathogenic variants were identified among the 244 inherited retinal disease-related genes. The possibility of pathological mutations not being assessed cannot be excluded.

## Discussion and conclusion

Heterozygous female carriers of XLRP may exhibit signs of distinctive mosaic retinopathy and variable phenotype. Several studies have identified some characteristics of mosaic retinopathies. However, most cases exhibit varying degrees of fundus changes with bilateral symmetry, and reports regarding XLRP carriers showing discordance are limited [11, 12, 15]. Furthermore, few studies have presented detailed multimodal imaging findings with functional assessments in this unique population. To our knowledge, the present study is the first to report a novel *RP2* mutation and to present the detailed multimodal imaging characteristics of an XLRP carrier patient, exhibiting remarkable asymmetry between the eyes.

Ophthalmologic findings in XLRP-carrier females can range from a tapetal-like reflex and isolated regions of peripheral pigment atrophy and clumping to extensive retinal degeneration, including diffuse bone spicule pigmentation and vessel attenuation [16]. Comander et al. [16] showed a wide range of functions among XLRP carriers, and most carriers had mildly or moderately reduced visual function but rarely became legally blind. Patients with *RP2* variants predominantly exhibit a RP phenotype. One study comparing the phenotypic features between XLRP revealed that, on average, visual acuity at all ages was lower in the *RP2* group than in the *RPGR* group [17]. This was likely due to early macular involvement in *RP2*. Jayasundera et al. [18] presented two cases of *RP2* female carriers with a phenotype similar to that of affected males. They exhibited atrophic macular changes, poor visual acuity, and central scotoma. The right eye of the patient in this study also manifested macular involvement, poor visual acuity, and central scotoma, which corresponds with the findings of previous reports. Jayasundera et al. [18] have also shown that one female carrier demonstrating asymmetrical disease had anisometropia of 8.00 D, the severely affected eye being myopic. In our patient, anisometropia of approximately 3.00 D, with the severely affected eye showing myopic tessellated fundus, further supported the association of myopia with *RP2* retinopathy. The mildly affected eye exhibited classic tapetal-like reflex with bright radial hyperautofluorescence in XLRP carriers on FAF images. FA and OCTA revealed no evidence of vascular compromise in the retina, and the visual function in the ERG or visual field was also not affected in the OS. However, the OCT findings from her mildly affected eye support previous reports of increased reflectivity from the RPE and irregularities or disruption of the ellipsoid zone band [19, 20]. In patients with inherited retinal disease and marked interocular asymmetry, microperimetry or multifocal ERG could be useful in detecting localized abnormalities in the better eye. Decaying visual function in the left eye over time may occur during long-term follow-up.

The phenomenon of XLRP patients exhibiting various phenotypes is commonly explained by mosaicism, which is the consequence of lyonization or random X-inactivation [10, 11]. During embryonic development, especially at the two-cell stage, a genetic and epigenetic mosaic embryo could be found because of asymmetric cell division [21]. Gametic half-chromatid mutations, mitotic selective chromatid segregation, chromosomal disjunction, asymmetric mitosis, and post-zygotic mutations are well-known mechanisms underlying this phenomenon [21]. As an embryonic left–right separation mechanism explained above occurs, mosaic embryos with different proportions of cells carrying disease-causing mutations on the left and right would develop into an individual with the disease manifesting asymmetrically on the left and right. In this case, we assumed that the proportion of cells carrying the wild-type X chromosome and those carrying the mutant X chromosome was different in the left and right eyes, from the mosaic embryonic stage. It is reasonable to hypothesize that the activation ratio of the X chromosome with the *RP2* gene mutation was be higher in the right eye in our case. Lyonization during clonal expansion early in photoreceptor cell differentiation and peripheral migration could result in mosaic patterns within the retina, random variation in the total amount of retinal tissue affected, and interocular differences in severity [12].

The *RP2* gene encodes the RP2 protein, which has two domains: an N-terminal tubulin folding cofactor C-like (TBCC) domain and a C-terminal nucleoside diphosphate kinase-like (NDPK) domain [13, 22]. RP2 acts as a GTPase-activating protein specifically for ADP-ribosylation factor like GTPase 3 (ARL3), which interacts with the N-terminal TBCC domain of the RP2 protein [23]. These proteins play an important role in the assembly and trafficking of membrane-associated proteins in the photoreceptor cilium [17, 24, 25]. *RP2* mutations include nonsense, missense, frameshift, insertion and deletion changes, which in most cases result in a severely truncated form of the protein [26–31]. In the current study, a novel mutation, c.803dup, p.Glu269Glyfs*7 of the *RP2* gene was identified, expanding the spectrum of *RP2* mutations that cause XLRP. The mechanism of premature termination of translation due to frameshift mutation would most likely result in the lack of translation of the RP2 protein. In cases where premature stop codons are located in the terminal exon, the truncation of the C-terminus of *RP2* results in a misfolded and nonfunctional protein. Rather than localizing to the plasma membrane, as is the case for the wild-type protein, it localizes to the cytoplasm and is susceptible to enhanced lysosomal degradation [32–34].

In conclusion, we have presented the detailed multimodal imaging analysis of an XLRP carrier female showing marked asymmetrical retinal involvement and identified a novel mutation in the *RP2* gene. Random X-inactivation may be attributed to interocular differences in severity in this unique population. A novel frameshift mutation in the *RP2* gene and a comprehensive phenotypic evaluation in the current study may broaden the spectrum of *RP2* mutations and the phenotypic spectrum of the disease in XLRP carriers.

## Supplementary Information

**Additional file 1.**

## Data Availability

All data generated or analyzed during this study are included in this published article.

## Abbreviations

RP: Retinitis pigmentosa
RPE: Retinal pigment epithelium
XLRP: X-linked retinitis pigmentosa
RPGR: Retinitis pigmentosa GTPase regulator
OD: Right eye
OS: Left eye
OCT: Optical coherence tomography
FAF: Fundus autofluorescence
FA: Fluorescein angiography
OCTA: Optical coherence tomography angiography
ERG: Electroretinography
NGS: Next-generation sequencing
ACMG: American College of Medical Genetics and Genomics
TBCC: Tubulin folding cofactor C-like
NDPK: Nucleoside diphosphate kinase-like
ARL3: ADP-ribosylation factor like GTPase 3
