# Supplementary material for: Asymmetric presentation with a novel RP2 gene mutation in X-Linked retinitis pigmentosa: a case report
Source: BMC Ophthalmol. 2023 May 17;23:221. doi: 10.1186/s12886-023-02968-4 (PMC10190057; doi:10.1186/s12886-023-02968-4)
Supplement: Supplementary file 1 — Additional file 1. [file 12886_2023_2968_MOESM1_ESM.docx]

**Supplementary Table 1.** Next-generation sequencing-based gene panel consisted of 244 genes associated with inherited retinal disease

| *ABCA4* | *ABCC6* | *ABHD12* | *ACO2* | *ADAM9* | *ADAMTS18* | *ADIPOR1* | *AGBL5* | *AHI1* | *AHR* |
| --- | --- | --- | --- | --- | --- | --- | --- | --- | --- |
| *AIPL1* | *ALMS1* | *ARHGEF18* | *ARL2BP* | *ARL3* | *ARL6* | *ATF6* | *ATXN7* | *BBIP1* | *BBS1* |
| *BBS10* | *BBS12* | *BBS2* | *BBS4* | *BBS5* | *BBS7* | *BBS9* | *BEST1* | *C12orf65* | *C1QTNF5* |
| *C21orf2* | *C2orf71* | *C8orf37* | *CA4* | *CABP4* | *CACNA1F* | *CACNA2D4* | *CAPN5* | *CC2D2A* | *CDH23* |
| *CDH3* | *CDHR1* | *CEP164* | *CEP250* | *CEP290* | *CERKL* | *CFH* | *CHM* | *CIB2* | *CLCC1* |
| *CLN3* | *CLRN1* | *CNGA1* | *CNGA3* | *CNGB1* | *CNGB3* | *CNNM4* | *COL11A1* | *COL2A1* | *COL9A1* |
| *CRB1* | *CRX* | *CSPP1* | *CTNNA1* | *CYP4V2* | *DFNB31* | *DHDDS* | *DHX38* | *DMD* | *DRAM2* |
| *EFEMP1* | *ELOVL1* | *ESPN* | *EYS* | *FAM161A* | *FLVCR1* | *FSCN2* | *FZD4* | *GDF6* | *GNAT1* |
| *GNAT2* | *GNB3* | *GNPTG* | *GPR125* | *GPR179* | *GPR98* | *GRK1* | *GRM6* | *GUCA1A* | *GUCA1B* |
| *GUCY2D* | *HARS* | *HGSNAT* | *HK1* | *HMCN1* | *HMX1* | *IDH3B* | *IFT140* | *IFT172* | *IFT27* |
| *IFT81* | *IMPDH1* | *IMPG1* | *IMPG2* | *INPP5E* | *INVS* | *IQCB1* | *ITM2B* | *JAG1* | *KCNJ13* |
| *KCNV2* | *KIAA0090* | *KIAA1549* | *KIF11* | *KLHL7* | *LAMA1* | *LCA5* | *LRAT* | *LRIT3* | *LRP5* |
| *LZTFL1* | *MAK* | *MAPKAPK3* | *MERTK* | *MFN2* | *MFRP* | *MFSD8* | *MKKS* | *MKS1* | *MTTP* |
| *MVK* | *MYO7A* | *NDP* | *NEK2* | *NEUROD1* | *NMNAT1* | *NPHP1* | *NPHP3* | *NPHP4* | *NR2E3* |
| *NRL* | *NYX* | *OAT* | *OFD1* | *OPA3* | *OPN1LW* | *OPN1MW* | *OTX2* | *PANK2* | *PAX2* |
| *PCDH15* | *PCYT1A* | *PDE6A* | *PDE6B* | *PDE6C* | *PDE6G* | *PDE6H* | *PDZD7* | *PEX1* | *PEX2* |
| *PEX7* | *PGK1* | *PHYH* | *PITPNM3* | *PLA2G5* | *PLK1S1* | *PLK4* | *PNPLA6* | *POC1B* | *POC5* |
| *POMGNT1* | *PRCD* | *PROM1* | *PRPF3* | *PRPF31* | *PRPF4* | *PRPF6* | *PRPF8* | *PRPH2* | *PRPS1* |
| *RAB28* | *RAX2* | *RB1* | *RBP3* | *RBP4* | *RD3* | *RDH11* | *RDH12* | *RDH5* | *REEP6* |
| *RGR* | *RGS9* | *RGS9BP* | *RHO* | *RIMS1* | *RLBP1* | *ROM1* | *RP1* | *RP1L1* | *RP2* |
| *RP9* | *RPE65* | *RPGR* | *RPGRIP1* | *RPGRIP1L* | *RS1* | *SAG* | *SAMD11* | *SDCCAG8* | *SEMA4A* |
| *SLC24A1* | *SLC25A46* | *SLC7A14* | *SNRNP200* | *SPATA7* | *SPP2* | *TEAD1* | *TIMP3* | *TMEM216* | *TMEM237* |
| *TOPORS* | *TREX1* | *TRIM32* | *TRNT1* | *TRPM1* | *TSPAN12* | *TTC8* | *TTLL5* | *TTPA* | *TUB* |
| *TUBGCP4* | *TUBGCP6* | *TULP1* | *UNC119* | *USH1C* | *USH1G* | *USH2A* | *VCAN* | *WDPCP* | *WDR19* |
| *WFS1* | *ZNF408* | *ZNF423* | *ZNF513* |  |  |  |  |  |  |
